# Process Parameter Prediction and Modeling of Laser Percussion Drilling by Artificial Neural Networks

**DOI:** 10.3390/mi13040529

**Published:** 2022-03-27

**Authors:** Chau-Shing Wang, Yang-Hung Hsiao, Huan-Yu Chang, Yuan-Jen Chang

**Affiliations:** 1 Department of Electrical Engineering, National Changhua University of Education, Changhua 50007, Taiwan; cswang@cc.ncue.edu.tw; 2 Department of Mechanical Engineering, National Yunlin University of Science and Technology, Douliou 64002, Taiwan; m10411010@yuntech.edu.tw (Y.-H.H.); m10811043@yuntech.edu.tw (H.-Y.C.)

**Keywords:** laser, percussion drill, blind hole, plasma, artificial neural network

## Abstract

Finding process parameters for laser-drilled blind holes often relies on an engineer’s experience and the trial-and-error method. However, determining such parameters should be possible using methodical calculations. Studies have already begun to examine the use of neural networks to improve the efficiency of this situation. This study extends the field of research by applying artificial neural networks (ANNs) to predict the required parameters for drilling stainless steel with a certain depth and diameter of blind holes, and it also pre-simulates the drilling result of these predicted parameters before actual laser processing. The pre-simulated drilling results were also compared with real-world observations after drilling the stainless steel. These experimental findings confirmed that the proposed method can be used to accurately select laser drilling parameters and predict results in advance. Being able to make these predictions successfully reduces time spent, manpower, and the number of trial-and-error shots required in the pre-processing phase. In addition to providing specific data for engineers to use, this method could also be used to develop a set of reference parameters, greatly simplifying the laser drilling process.

## 1. Introduction

Laser processing has the characteristics of high speed and stable energy. These properties have led to the widespread adoption of laser processing in the manufacturing, automotive, and electronics industries. Lasers can be applied in various ways, including cutting specific shapes on diamonds [1], material removal on CBN (cubic boron nitride) [2], drilling YSZ (yttrium stabilized zirconia) materials [3], micro-drilling aluminum materials [4], drilling holes without creating cracks on surfaces [5], and sintering materials [6]. However, controlling the depth of blind holes in laser drilling remains a challenge. In fact, the depth of laser-drilled holes is affected by many factors, such as the energy of the laser pulse, the pulse frequency, properties of the material being worked, spatter, and gas pressure. Controlling high-aspect-ratio blind holes with small diameters represents even more of a challenge, as accurately controlling the depth of blind holes in laser drilling requires a substantial amount of effort [7]. Daniel et al. [8] presented a mathematical model to estimate the drilled hole depth based on the homogenous distribution radiation of the laser on the wall of the drilled holes. However, it must be assumed that the drilled hole is conical, and it might not be suitable in cases full of variables. Numerical simulation techniques have also been applied to predict the depth and processing time in the laser drilling process. Meshless and particle-based methods were suitable for the simulation of the laser ablation process regarding its material loss in the process. The smoothed particle hydrodynamics (SPH) modeling technique was used to model the transient heat conduction during the interaction between the laser and material [9]. In order to improve the robustness and efficiency of the computational calculation, several techniques have been developed, such as the particle strength exchange (PSE) method [10], the symmetric smoothed-particle hydrodynamics (SSPH) method [11], or the meshless local Petrov–Galerkin (MLPG) collocation method [12]. A three-dimensional simulation of the laser drilling process by meshless methods with six different schemes was performed to compare runtimes at three different resolutions of particles [13]. With numerical simulation techniques, however, process variables, for example, absorptivity of material, latent heat, or assist gas, were ignored in the computational modeling.

Neural networks can construct nonlinear models, can accept different kinds of variables as input, and have strong adaptability. They have been used for prediction, modeling, and parameter optimization in various laser processing techniques in recent years. One study indicated that artificial intelligence could extract knowledge concealed in experimental data, so that complex decisions could be made without manual intervention [14]. In 2003, Yousef employed a neural network to predict laser energy using just the material type, drilling depth, and diameter for single-shot laser processing as inputs. This investigation demonstrated that neural networks can be used to predict the required processing parameters [15].

Since artificial neural networks (ANNs) can only determine parameter conditions through constant recalculation, they may not provide the best processing solution. For this reason, Solati et al. [16] combined an ANN with a genetic algorithm (GA), using the ANN to process the relationship equation first for material removal, and then using the GA to optimize the parameters. Sibalija et al. [17] proposed a method of integrating ANNs with GA and compared this design with Taguchi’s quality loss function. Experimental laser drilling results showed that the integration of an ANN generated a system that could find a global solution within a continuous space.

In addition to optimizing ANNs, a GA can also be used alone to generate predictive algorithms. In one study, a GA was employed to establish multi-gene genetic programming (MGGP). Subsequently, scholars compared outcomes between ANNs when combined with MGGP, predicting the strength and processing time of processed graphene sheets using processing temperature, drill speed, and drill feed. Their results showed that MGGP-enhanced ANNs significantly outperformed conventional ANNs [18].

Patel et al. [19] compared the accuracy of fuzzy logic, regression models, and ANNs when predicting groove widths in laser-cut, glass-fiber-reinforced polymers. As input parameters, they tested changes in laser power, auxiliary gas pressure, and cutting speed. Mustafa et al. [20] compared the results of extreme learning machines (ELMs) with ANNs to predict droplet spatter area, hole diameter, and hole inclination for the laser drilling of a titanium alloy, a material commonly used in aerospace engineering. In this study, not only was the ELM model more accurate than the ANN, but its operation time was shorter.

Another study incorporated analysis of variance into the response surface methodology (RSM) to enhance prediction accuracy, and then compared it with a feed-forward, back-propagation ANN to predict groove depth, groove width, and pattern similarity for machined Al–SiC composites. The input parameters comprised auxiliary gas flow, focus position, pulse frequency, and laser current. Overall, the ANN demonstrated a more uniform performance [21]. Dhaker et al. [22] used an adaptive neuro-fuzzy inference system that combined a GA and fuzzy logic to optimize parameters. It achieved accurate results by governing control parameters.

Most of the study works focused on predicting process parameters or predicting drilling depth and geometry, separately. Thus, this study proposes a complete architecture combining process parameter prediction and processing result simulation. With the proposed method, the operator only needs to input the desired drilling depth and hole diameter, and then the system can predict the required process parameters. At the same time, the proposed system can also simulate the processing results, and the operator can check whether the simulation error is within the acceptable range as the basis for whether to adjust the parameters. Through such a pre-simulation, the number of trial parameters can be reduced and the preparation time of finding parameters can be shortened. The quality of the drilled holes can also be confirmed in advance.

## 2. Pre-Processing of the Experiment

**Figure 1:**
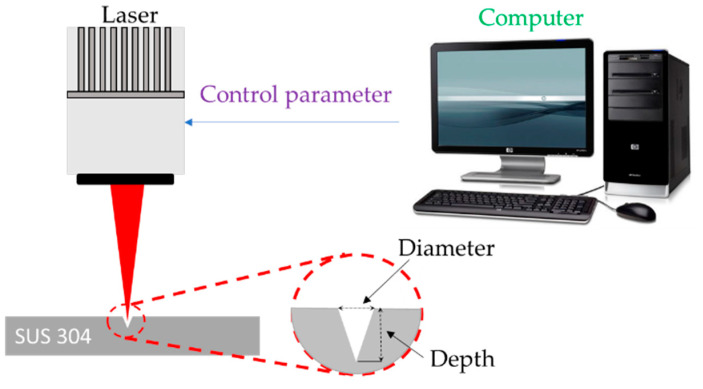
Schematic figure of laser drilling.

The laser source in this study was a 20-W EP-Z Pulsed Fiber Laser (PFL). Detailed specifications of the experimental setup are presented in Table 1. A schematic figure of laser drilling is shown in Figure 1. The processing material selected for testing was 304 stainless steel, 50 mm × 50 mm × 2 mm with a surface roughness Ra of 0.07. Table 2 shows the precise material composition provided by the stainless steel manufacturer.

Before processing, an ultrasonic vibration machine was used to clean the surface of the material with acetone and ethanol for 20 and 25 s, respectively. The focus position was located on the surface of the material. Each hole’s drilling location was located 0.5 mm apart. To ensure experimental reliability and reproducibility, the position of the material and the z-axis of the laser processing machine were not adjusted when the material was placed onto the laser machining platform.

### 2.1. Experimental Data Collection

Many factors affect laser processing results, including laser pulse width, pulse repetition frequency, pulse waveform, pulse energy, number of pulses, and auxiliary gas pressure. The amount of laser energy applied to the material surface directly affects the laser drilling depth; therefore, among these factors, the combination of laser pulse energy and number of pulses is the most direct factor affecting drilling depth. Therefore, this study selected these two factors as the key variables of interest and held other parameters fixed for the duration of the experiment. The laser pulse energy settings used in this study were 0.45, 0.475, and 0.5 mJ. The number of shots used ranged from 1 to 40. Each combination of shots at each energy level were performed nine times for reliability, thus obtaining 1080 sets of data. Each data set contained the laser pulse energy, number of shots, drilled-hole diameter, and depth.

### 2.2. Measurement of Hole Diameter and Depth

**Figure 2:**
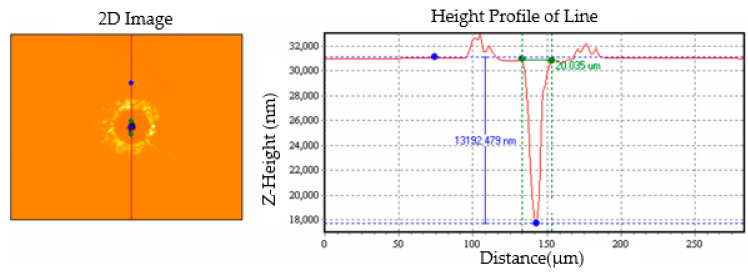
Depth measurement using the white-light interferometer.

In order to reduce any errors caused by measurement and to ensure that the measurements were as accurate as possible, we used two types of instruments to measure the diameter and depth of the drilled holes. The first instrument was a SWIM-1510MS white-light interferometer obtained from Taizhi Precision Technology Company. It has a measurement accuracy of 1 nm increments and was used to measure the depth of shallow drilled holes. The measurement method is illustrated in Figure 2.

**Figure 3:**
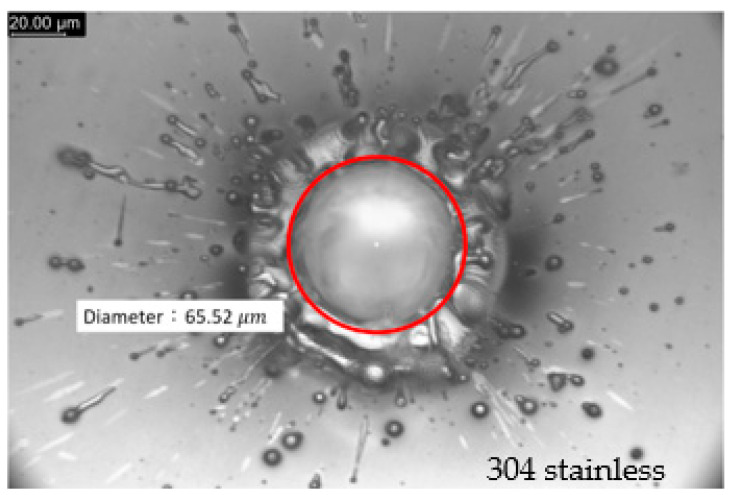
Diameter measurement using a microscopy (with laser drilling parameters of 0.5 mJ laser pulse energy and 12 laser pulse shots).

Where the number of shots exceeded 10, the drilled-hole depth could not be measured accurately with a white-light interferometer. Therefore, a microscopy with an Olympus U-PMVTC was used in focus mode with a resolution of 0.5 μm. By using this instrument, the hole diameters were also measured from the captured images, as shown in Figure 3.

## 3. Methodology

**Figure 4:**
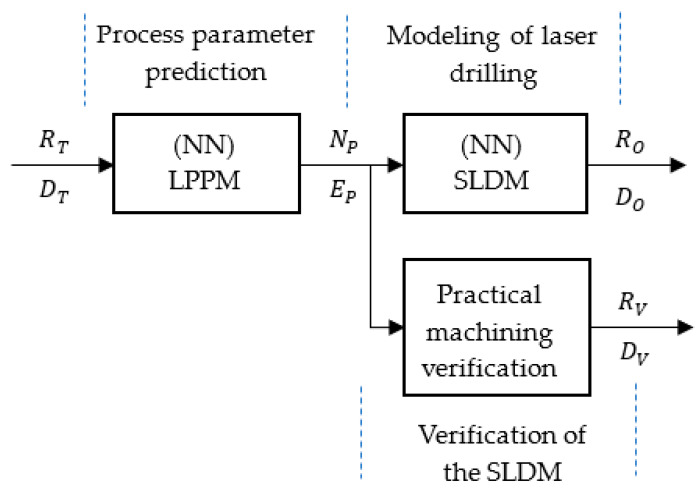
Methodological flow chart for parameter prediction and modeling of laser drilling, and its verification.

This study established two neural network models based on the deep learning toolbox in MATLAB and combined these with the practical laser machining process shown in Figure 4. First, the target diameter *R_T_* and target depth *D_T_* data were fed into the laser parameter prediction model (LPPM). The number of laser shots *N_P_* and the laser pulse energy *E_P_* required were given in the output. Second, the LPPM output was then input to the simulated laser drilling model (SLDM) to simulate the drilling results and provide the values of the simulated hole diameter *R_O_* and depth *D_O_*. Third, the LPPM-predicted *N_P_* and *E_P_* values were verified by actual laser machining, whereupon the drilled-hole diameters and hole depths, *R_V_* and *D_V_*, respectively, were recorded for verification.

To build the models of LPPM and SLDM, 70% of the 1080 data sets were used for training and 30% were used for testing. The LPPM and SLDM were trained separately.

### 3.1. LPPM

**Figure 5:**
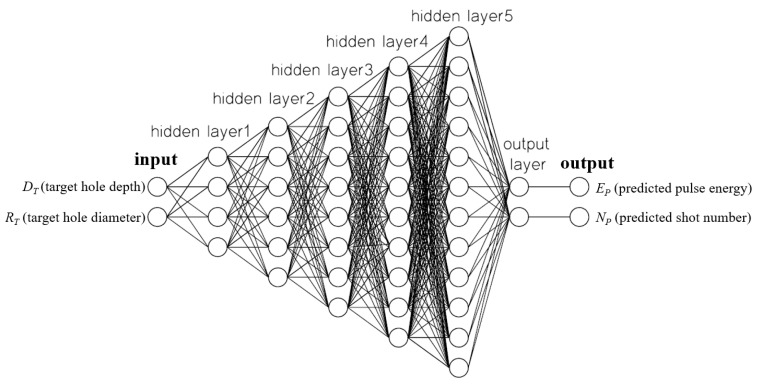
ANN-based LPPM for parameter prediction.

The LPPM was used to predict the laser pulse energy *E_P_* and number of laser shots *N_P_* required for drilling a hole of a certain size. Therefore, the inputs of the LPPM were hole diameter and depth, and the outputs were laser pulse energy and number of shots. In order to find suitable numbers of hidden layers and neurons of the LPPM, the following trials were conducted and the optimal one was selected. As shown in Table 3, group A had five hidden layers and the number of neurons in each layer were 4, 6, 8, 10 and 12. Group B had four hidden layers and the number of neurons in each layer were 2, 5, 6 and 8. Group C had three hidden layers and the number of neurons in each layer were 2, 5 and 6. After comparing the results of using three, four, and five hidden layers, a five-layer structure was selected because its error value was the smallest, as shown in Table 3. In order, the hidden layers of the LPPM contained 4, 6, 8, 10, and 12 neurons. The output layer contained two neurons, and as Figure 5 shows, the network type was feed-forward, back-propagation.

### 3.2. LPPM Training with Five-fold Cross-Validation

The 1080 data entries were sorted, randomly numbered, and divided into 5 groups of 216 entries. The group order was changed, and they were fed into the ANN for calculation and analysis. Table 4 shows that there was no particularly beneficial performance for a given combination, implying that there was no specific pattern to the data. 

**Figure 6:**
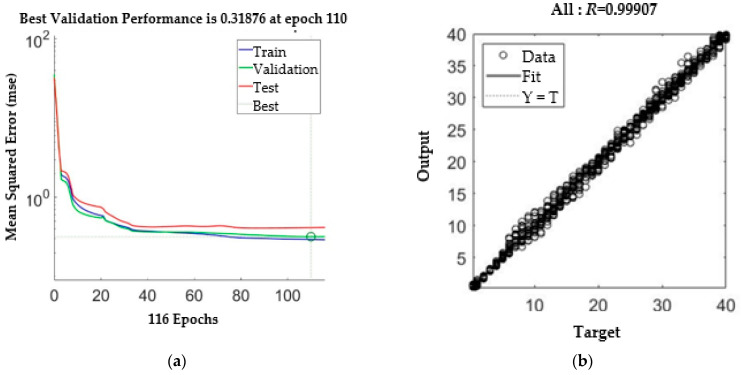
LPPM training. (**a**) MSE. (**b**) Regression results.

The group with the smallest mean square error (MSE) of approximately 0.325 was selected for use in subsequent experiments. During the training process, and using the groups, the minimal MSE was arrived at on the 110th iteration. The *R* value was 0.999, as shown in Figure 6.

### 3.3. SLDM

**Figure 7:**
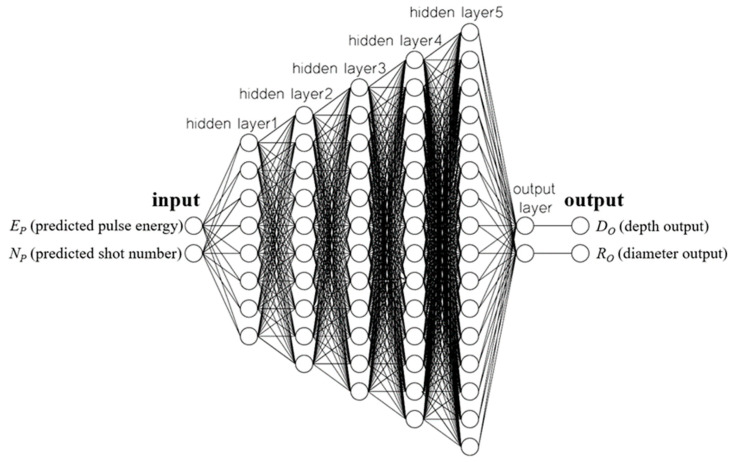
ANN-based SLDM for drilling simulation.

**Figure 8:**
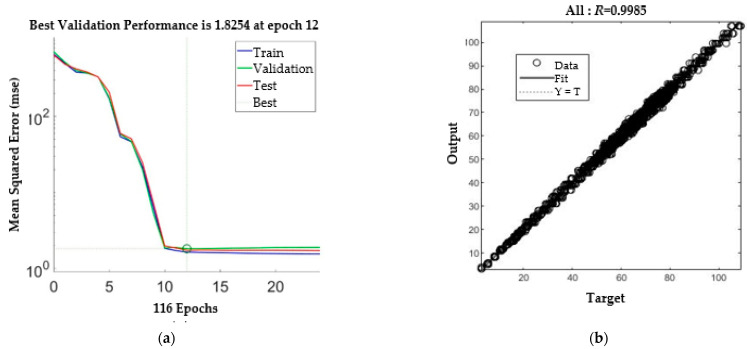
SLDM training. (**a**) MSE. (**b**) Regression results.

The SLDM was the second ANN and was used to simulate the laser drilling results. The inputs for the SLDM were the number of shots and laser pulse energy, and outputs for the SLDM were hole diameter and depth. Through the same attempt as with the LPPM, the model of SLDM chose five hidden layers, each with 8, 10, 12, 14, and 16 neurons, respectively, as shown in Figure 7. The MSE obtained after training was 1.8254 and the *R* value was 0.9985, as displayed in Figure 8.

## 4. Experimental Results

### 4.1. LPPM

For training the LPPM, 70% of the 1080 data entries was used and 30% of the data were for testing. When the training was completed, the average error of *E_P_* was 0.27% and the average error of *N_P_* was 0.43%. On the other hand, for testing, the average errors for *E_P_* and *N_P_* were 0.52% and 0.57%, respectively, as shown in Table 5. One of the sources of these errors is explained below.

The output of predicted shot number by the LPPM may be a number with decimals, but the shot number should be an integer number. Thus, the predicted value was rounded to an integer. As a result, there is a gap visible between the actual and predicted values. The effects of rounding are discussed below. As Table 6 indicates, the initially predicted number of shots for data entry 1 was *N_Pi_* = 13.69. Rounding this to the nearest integer *N_P_* = 14 results in the actual diameter and depth (*R_V_* and *D_V_*) exceeding the target values (*R_T_* and *D_T_*). For data entry point 9, the *N_Pi_* was 10.39, and after rounding, *N_P_* = 10. The difference resulted in *R_V_* and *D_V_* being smaller than *R_T_* and *D_T_*, respectively. The table shows that when (*N_P_*−*N_Pi_*) is positive, the actual drilling results *R_V_* and *D_V_* are both larger than the target values *R_T_* and *D_T_*, respectively, where the errors are positive. When (*N_P_*−*N_Pi_*) is negative, *R_V_* and *D_V_* are both smaller than *R_T_* and *D_T_*, respectively, and the errors are negative.

**Figure 9:**
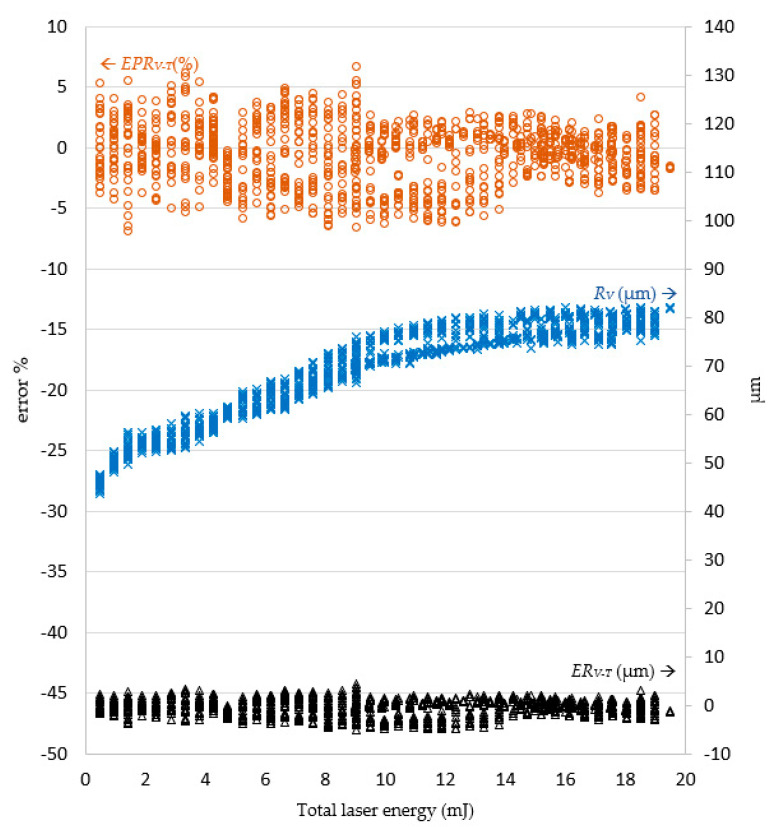
The error *ER_V−T_* between the target and actual hole diameter (*R_T_* and *R_V_*) corresponds to the total laser energy. Note: *ER_V−T_* = *R_V_ − R_T_*; *EPR_V−T_* = (*R_V_ − R_T_)/R_T_* (%).

**Figure 10:**
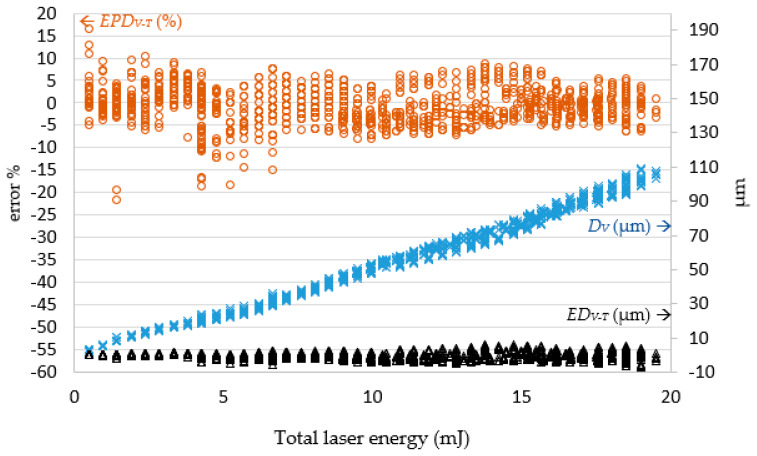
The error *ED_V−T_* between the target and actual hole depth (*D_T_*, *D_V_*) corresponds to the total laser energy. Note: *ED_V−T_* = *D_V_ − D_T_*; *EPD_V−T_* = (*D_V_ − D_T_)/D_T_* (%).

In the experiments, it was revealed that the laser pulse energy and number of laser shots predicted by the LPPM did not correspond to those in the original training data, but the target drilling results can still be achieved using the parameters predicted by the LPPM. For example, the training set contained two records *A1* (*R_T_*, *D_T_*, *N*, and *E*) = (76.93, 53, 21, and 0.5) and *A2* (*R_T_*, *D_T_*, *N*, and *E*) = (77.1, 53.5, 22, and 0.45). Here, *N* and *E* refer to the required number of shots and laser pulse energy, respectively. The target values predicted for *A1* and *A2* are very similar, despite the different *N* and *E* values. Because the total laser energy (*E_all_* = N*E) for *A1* and *A2* is the same, the predicted laser drilling results with either *A1* or *A2* parameters are similar. In order to verify the feasibility of the predicted pulse energy and number of shots, *E_P_* and *N_P_* were used as inputs for the laser machine for actual drilling. The results were then compared with the target value and the findings are shown in Figure 9 and Figure 10.

In Figure 9, to facilitate further examination of the relationship between the target diameter *R_T_* and actual diameter *R_V_*, the number of laser shots multiplied by the laser pulse energy were shown as total laser energy on the horizontal axis. The error is displayed on the left vertical axis, and the right vertical axis is the diameter on micrometers. When the total laser energy was smaller, the diameter was also smaller. As total laser energy increased, the diameter also increased. When the total laser energy was 10 mJ, the diameter value became stable and converged to 81.77 μm. When the total laser energy was less than 9 mJ, the percentage error on the diameter value ranged between 6.86% and −6.87%. When the total laser energy reached 14 mJ, the percentage error gradually declined to between 3.71% and −4.11%. Regardless of the total laser energy, the error magnitude remained less than 5 μm.

Figure 10 shows the error between the target depth *D_T_* and actual depth *D_V_*. When total laser energy was increased, the hole depth increased proportionally. When the total laser energy was less than 4 mJ, the processing hole depth error was between 1.27 and −1.44 μm. After the total energy reached 4 mJ, the depth error increased with total laser energy, converging on the range 6.77 to −5.83 μm. Once the total laser energy reached 7 mJ, the depth error remained within ± 10%.

The percentage error decreases gradually as the total laser energy increases. The experimental results show that the actual drilled diameter and depth errors based on the predicted parameters by the LPPM were gradually stabilized in relation to total laser energy.

### 4.2. SLDM

**Figure 11:**
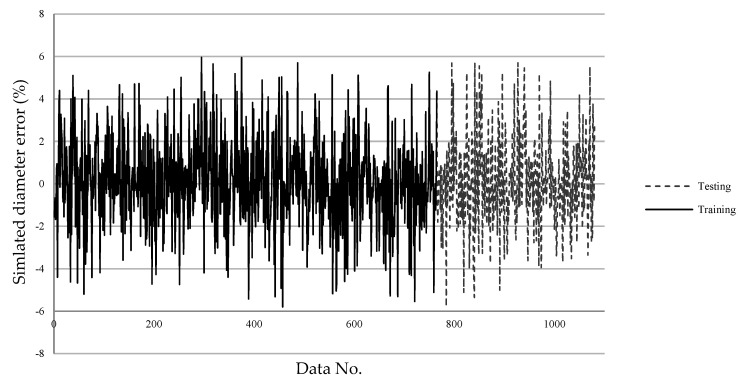
The SLDM-simulated diameter *R_O_* percentage error.

**Figure 12:**
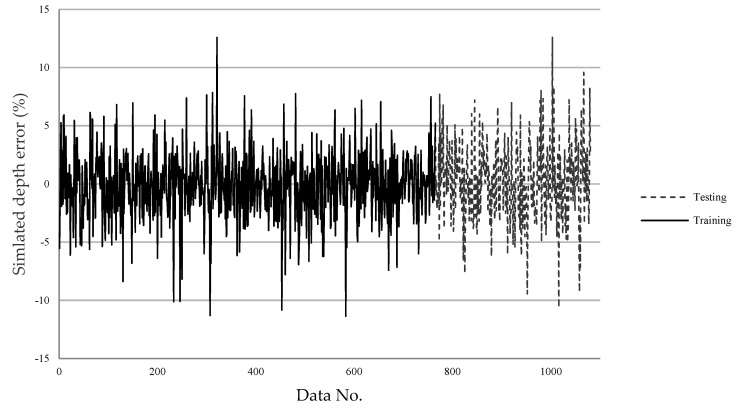
The SLDM-simulated depth *D_O_* percentage error.

The errors of the machining results simulated by the SLDM according to the predicted machining parameters by the LPPM are shown in Figure 11 and Figure 12. The final training errors are shown in a solid line from the 1st through 765th data entries, and the testing errors are shown in a dashed line from the 766th through 1080th data entries. The average error in the diameter value as simulated by the SLDM was 0.05 μm, and the average percentage error was 0.1%. The average error in magnitude and percentage for simulated depth were 0.07 μm and 0.14%, respectively. In testing, the magnitude of average error and percentage error for diameter were 0.67 μm and 1.25%, respectively. The magnitude of average error and percentage error for depth were 0.04 μm and 1.06%, respectively. Overall, the error in both diameter and depth values was less than 1.5%, as shown in Table 7. As displayed in Figure 11, the maximal simulated diameter error did not exceed 6%. In Figure 12, eight sets of parameters had a percentage error exceeding 10%; however, these sets only required two shots, which resulted in a shallow depth and a large percentage error.

**Figure 13:**
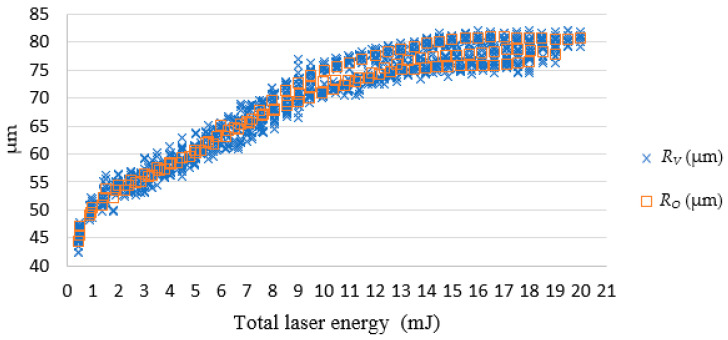
The SLDM-simulated *R_O_* and the actual diameter *R_V_*.

**Figure 14:**
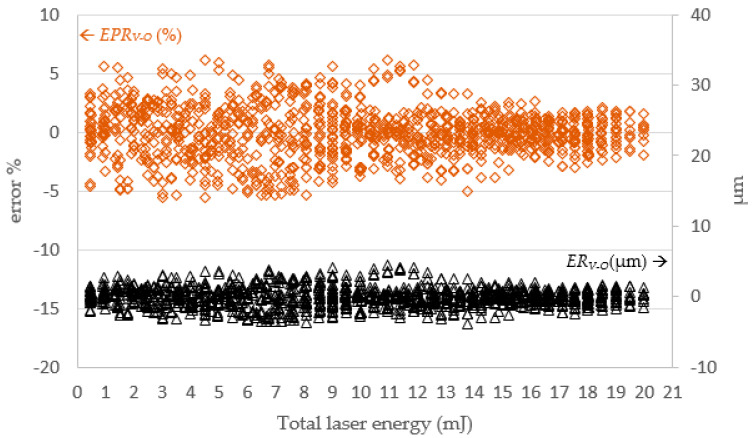
The SLDM-simulated diameter error versus total laser energy. Note: *ER_V−O_* = *R_V_ − R_O_*; *EPR_V−O_* = (*R_V_ − R_O_)/R_O_* (%).

In Figure 13, the simulated diameter *R_O_* and actual diameter *R_V_* were very close. When the total laser energy was less than 10 mJ, the drilled diameter value was proportional to total laser energy. When the total laser energy exceeded than 10 mJ, the drilled diameter tended to be stable. Figure 14 presents a comparison of *R_O_* and the *R_V_*. The simulated and actual values were similar and in favorable agreement, and the depth was proportional to the total energy of the laser.

**Figure 15:**
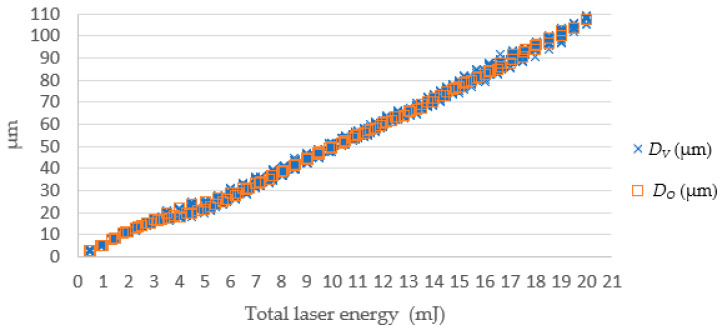
The SLDM-simulated *D_O_* and the actual depth *D_V_* values.

**Figure 16:**
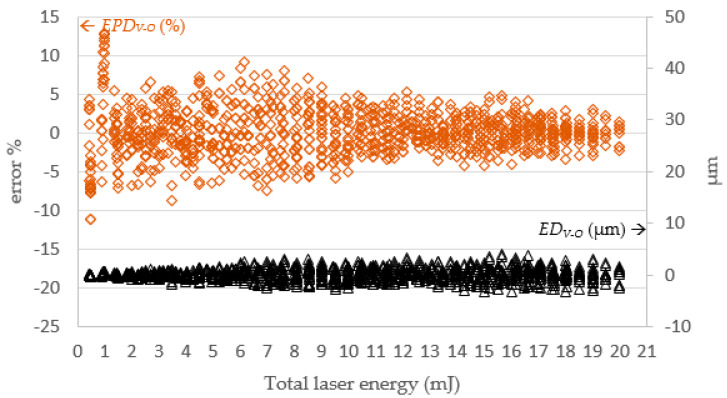
The SLDM-simulated depth error versus total laser energy. Note: *ED_V-O_* = *D_V_* − *D_O_*; *EPD_V-O_* = (*D_V_* − *D_O_*)/*D_O_* (%).

Figure 15 and Figure 16, respectively, show the simulated depth *D_O_*, actual depth *D_V_*, and the errors between them. The error magnitude was eventually stabilized and stayed within ± 5 μm. The percentage error declined with the total laser energy. This means the proposed SLDM has a good simulation ability for laser processing with low errors for deep drilled holes.

### 4.3. Combination of the LPPM and SLDM

**Figure 17:**
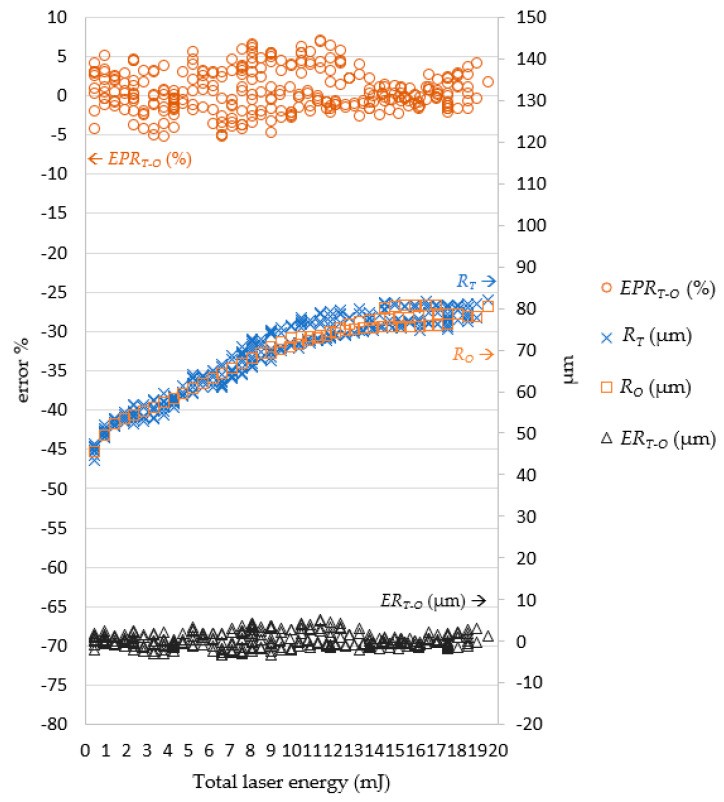
Comparison of *R_T_* and *R_O_*. Note: *ER_T__-O_* = *R_T_* − *R_O_*; *EPR_T__-O_* = (*R_T_* − *R_O_*)/*R_O_* (%).

**Figure 18:**
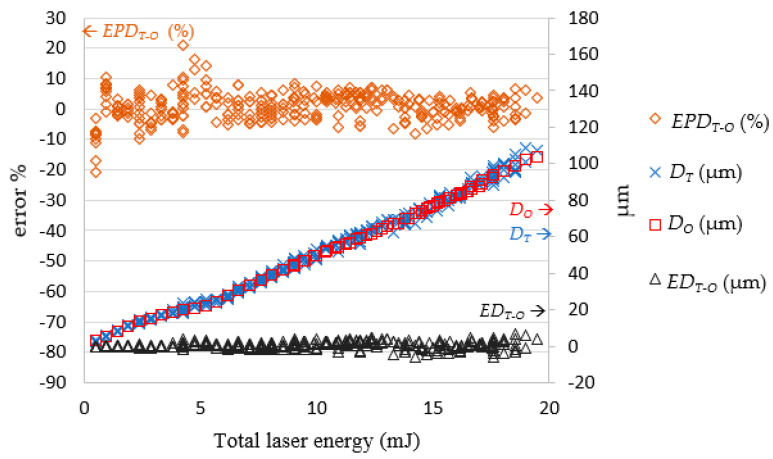
Comparison of *D_T_* and *D_O_*. Note: *ED_T-O_* = *D_T_* − *D_O_*; *EPD_T-O_* = (*D_T_* − *D_O_*)/*D_O_* (%).

After the LPPM and SLDM were established, the testing data was imported into the LPPM to generate the *E_P_* and *N_P_* values. Then, the data was passed to the SLDM for laser drilling simulation, which produced the *R_O_* and *D_O_* values. As shown in Figure 17, the SLDM-simulated diameter *R_O_* was extremely close to the target diameter *R_T_*. The magnitude and percentage error for the diameter readings were within ± 5.5 μm and ± 6.5%, respectively. As illustrated in Figure 18, the SLDM-simulated depth *D_O_* had good simulation and was very close to *D_T_*. The magnitude of error for the depth readings was within ± 6.8 μm. Through these tests, the proposed combination of the LPPM and SLDM models was shown to successfully predict the required laser processing parameters for holes of a targeted depth and diameter. It was also possible to quickly simulate laser drilling results by the SLDM when given the predicted process parameters in order to check the prediction accuracy of the LPPM in advance.

### 4.4. Parameter Verification

**Figure 19:**
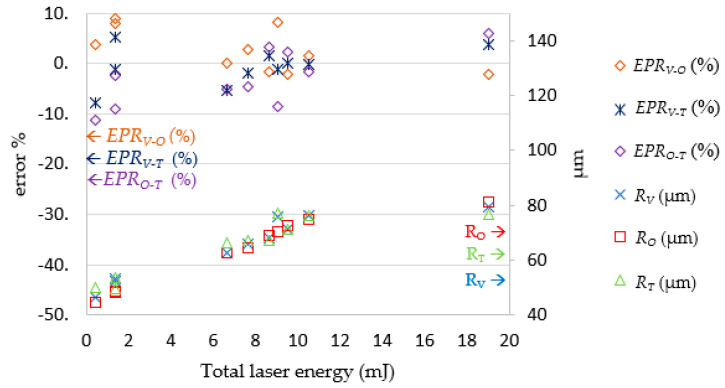
Comparison of *R_V_*, *R_T_*, and *R_O_*. Note: *EPR_V-O_* = (*R_V_* − *R_O_*)/*R_O_* (%); *EPR_V-T_* = (*R_V_* − *R_T_*)/*R_T_* (%); *EPR_O-T_* = (*R_O_* − *R_T_*)/*R_T_* (%).

**Figure 20:**
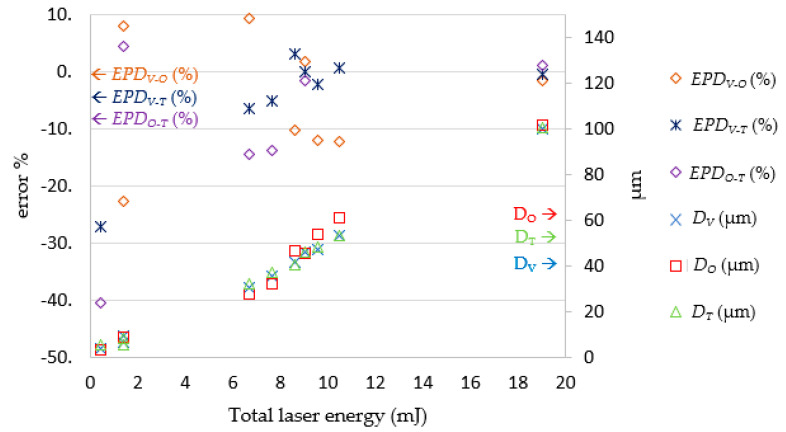
Comparison of *D_V_*, *D_T_*, and *D_O_*. Note: *EPD_V-O_* = (*D_V_ −* *D_O_*)/ *D_O_* (%); *EPD_V-T_* = (*D_V_* − *D_T_*)/*D_T_* (%); *EPD_O-T_* = (*D_O_ −* *D_T_*)/*D_T_* (%).

To determine whether the findings in the theoretical part of this study were suitable for real-world application, 10 sets of data were selected from the original testing data entries. Based on these 10 sets of data, the laser process parameters predicted by the LPPM were simulated by the SLDM and the simulation results were also verified and compared with outcomes from actual laser processing experiments. The experimental results and errors are presented in Table 8 and Figure 19 and Figure 20. They show that the actual hole diameter and depth were subject to a machining error of approximately 10 μm and 5 μm, respectively. These findings are in line with those of the previous experiment; that is, the percentage error was larger when total laser energy was lower. The reason for this phenomenon is that the LPPM output used to derive the number of shots uses a decimal system, which requires rounding and causes some errors.

## 5. Conclusions

This paper has presented an ANN-based laser process parameter prediction and drilling simulation system. The system was able to recommend parameters for laser drilling on 304 stainless steel and it was able to simulate the laser drilling results, i.e., hole depth and diameter. The magnitude of error for diameter measurements was within ± 5.5 μm or ± 6.5%, and error for depth was within ± 6.8 μm. The depth percentage error was lower when total laser energy was larger; at energy values above 7 mJ, the depth error remained within ± 10%. This system can be used as an auxiliary tool for laser operators to adjust processing parameters. It can predict the result of drilling stainless steel in advance, and then the operator can increase or decrease the parameters according to the predicted value, which can reduce the time spent on adjusting the processing parameters.

## Figures and Tables

**Table 1:** Laser source information.

Laser Specifications	
Model	SP-020P-A-EP-S-A-Y
Laser module dimensions	347 × 201 × 95 (H,W,D) mm
Nominal average output power	20 W
Central emission wavelength	1059–1065 nm
Pulse repetition frequency (PRF) range	1–1000 kHz
Beam quality factor (M^2^)	1.3
Pulse duration range	3–500 ns

**Table 2:** 304 stainless steel material properties.

SUS304	Fe	C	Mn	P	S	Si	Cr
Percentage (%)	78.85	0.08	2.00	0.045	0.03	1.00	18.00
**Properties**	**Density (g/cm^3^)**		**Thermal Conductivity (W/m-°C)**		**Specific Heat (J/g-°C)**		**Melting Point (°C)**
Value	7.93		16.3 @ 100 °C		0.50		1398

**Table 3:** Errors as they varied by number of hidden layers.

Item		A	B	C
Training	*E_P_* error (%)	−0.28	0.25	0.25
	*N_P_* error (%)	0.79	2.36	1.89
Testing	*E_P_* error (%)	−0.53	0.04	0.04
	*N_P_* error (%)	1.23	3.58	2.75

A: Five hide-layer, neuron numbers 4, 6, 8, 10, 12; B: four hide-layer, neuron numbers 2, 5, 6, 8; and C: Three hide-layer, neuron numbers 2, 5, 6.

**Table 4:** Neural network five-fold cross-validation results.

Data Order:		VWXYZ	WXYZV	XYZVW	YZVWX	ZVWXY
Training	*E_P_* error (%)	−0.28	0.14	0.53	0.18	−0.20
	*N_P_* error (%)	0.79	0.74	0.74	0.39	0.29
Testing	*E_P_* error (%)	−0.53	0.01	0.69	0.20	0.17
	*N_P_* error (%)	1.23	0.37	0.37	0.65	−0.23
Mean-Square Error		0.325	0.34	0.423	0.331	0.40

V: Data No. 001–No. 216; W: Data No. 217–No. 432; X: Data No. 433–No. 648; Y: Data No. 649–No. 866; and Z: Data No. 867–No. 1080.

**Table 5:** Average error of training and testing for the LPPM.

Item		Average Error (%)
Training	Predicted energy *E_P_*	0.27
	Predicted shot no. *N_P_*	0.43
Testing	Predicted energy *E_P_*	0.52
	Predicted shot no. *N_P_*	0.57

**Table 6:** Errors when rounding to produce the number of shots required.

Data Entry:		1	2	3	4	5	6	7	8	9	10
Target	*R* *_T_*	64.07	76.19	63.35	62.46	57.30	79.61	76.22	63.08	61.39	73.55
	*D*_*T*_	29.50	68.50	26.00	23.50	18.68	71.00	71.00	23.50	21.50	62.00
Predicted parameters	*E*_*P*_	0.50	0.475	0.475	0.45	0.45	0.475	0.475	0.45	0.45	0.475
Without rounding:	*N*_*Pi*_	13.69	29.73	12.37	11.34	8.17	28.14	30.90	11.38	10.39	26.91
Rounding:	*N*_*P*_	14.00	30.00	12.00	11.00	8.00	28.00	31.00	11.00	10.00	27.00
Actual	*R*_*V*_	65.96	78.86	59.61	57.98	57.23	77.95	76.47	61.95	58.43	75.86
	*D*_*V*_	31.00	71.00	25.50	20.00	17.94	67.00	74.50	22.50	19.20	63.00
Errors	*N_P_-* *N_Pi_*	+0.31	+0.27	−0.37	−0.34	−0.17	−0.14	+0.10	−0.38	−0.39	+0.09
	*ER* *_V-T_*	1.89	2.67	−3.74	−4.48	−0.07	−1.66	0.25	−1.13	−2.96	2.31
	*ED* *_V-T_*	1.50	2.50	−0.50	−3.50	−0.75	−4.00	3.50	−1.00	−2.30	1.00

**Table 7:** Training and testing error for the SLDM.

Item		Average Error (%)
Training	Simulated diameter *R_O_*	0.1
	Simulated depth *D_O_*	0.14
Testing	Simulated diameter *R_O_*	1.25
	Simulated depth *D_O_*	1.06

**Table 8:** Parameter verification results.

Item	*R_T_*	*D_T_*	*E_P_*	*N_Pi_*	*N_P_*	E_P_*N_P_	*R_O_*	*D_O_*	*R_V_*	*D_V_*	Error (%)					
											*ER_V-O_*	*ED_V-O_*	*ER_V-T_*	*ED_V-T_*	*ER_O-T_*	*ED_O-T_*
1	49.50	5.59	0.46	2.65	3.00	1.39	48.32	8.80	52.10	6.80	7.82	−22.73	5.25	21.65	−2.39	57.44
2	50.08	5.52	0.45	1.13	1.00	0.45	44.46	3.28	46.16	4.02	3.81	22.52	−7.83	−27.17	−11.21	−40.56
3	53.61	8.30	0.46	3.32	3.00	1.39	48.68	8.68	53.03	9.37	8.93	7.95	−1.08	12.83	−9.19	4.52
4	66.05	32.33	0.48	13.52	14.00	6.67	62.58	27.65	62.57	30.25	−0.03	9.39	−5.28	−6.43	−5.25	−14.47
5	67.04	40.50	0.48	18.16	18.00	8.62	69.18	46.51	68.03	41.75	−1.66	−10.23	1.47	3.09	3.19	14.83
6	67.35	37.17	0.48	15.57	16.00	7.64	64.28	32.03	66.01	35.25	2.68	10.06	−1.99	−5.17	−4.55	−13.83
7	76.92	46.00	0.48	18.56	19.00	9.05	70.23	45.26	75.92	46.00	8.10	1.65	−1.30	0.00	−8.69	−1.62
8	76.32	53.17	0.48	21.77	22.00	10.51	74.98	61.04	76.18	53.50	1.60	−12.35	−0.19	0.62	−1.76	14.80
9	76.58	100.50	0.49	38.75	39.00	19.04	81.22	101.69	79.45	100.00	−2.19	−1.66	3.74	−0.50	6.06	1.18
10	71.13	48.33	0.48	20.13	20.00	9.56	72.72	53.74	71.20	47.25	−2.09	−12.07	0.10	−2.23	2.24	11.19
